# Environmental Impact of Physical Visits and Telemedicine in Nursing Care at Home: Comparative Life Cycle Assessment

**DOI:** 10.2196/67538

**Published:** 2025-04-04

**Authors:** Egid M van Bree, Lynn E Snijder, Hans C Ossebaard, Evelyn A Brakema

**Affiliations:** 1 Department of Public Health and Primary Care Leiden University Medical Center Leiden The Netherlands; 2 Department of Surgery Maastricht University Maastricht The Netherlands; 3 Center for Sustainable Healthcare Amsterdam University Medical Centers Amsterdam The Netherlands; 4 Zorginstituut Nederland Diemen The Netherlands; 5 Athena Institute Vrije Universiteit Amsterdam Amsterdam The Netherlands; 6 National eHealth Living Lab Leiden University Medical Center Leiden The Netherlands

**Keywords:** carbon footprint, eHealth, telemedicine, telehealth, sustainable health care, digital health care, environmental impact, environment, physical visits, telemedicine, nursing, life cycle assessment, life cycle, ecology, sustainability, footprint, planetary health

## Abstract

**Background:**

The health care sector contributes notably to environmental harms, impacting human and ecosystem health. Hence, countries increasingly set ambitions to transition to environmentally sustainable health care, focusing on resource use, energy consumption, and patient travel. Telemedicine is often considered a promising solution to reduce travel-related carbon emissions. However, underlying environmental impact assessments lack important components such as staff travel and fail to adhere to standardized conduct and reporting. Moreover, assessments of telemedicine use in primary care are scarce.

**Objective:**

This study aims to quantify and compare the environmental impact of physical visits and telemedicine visits in the context of domiciliary care and home nursing.

**Methods:**

We conducted a life cycle assessment following international ISO-14040/44 standards of all resources required per individual patient visit, either in person at the patient’s home or via video calling with a dedicated user-friendly tablet. We collected anonymous user data in collaboration with a telemedicine service company, complemented by consulting staff members of four nursing organizations. Telemedicine visits were elementary in nature, such as supporting patients in taking their medication or structuring their daily agenda. We quantified average environmental impacts from cradle to grave, using the Environmental Footprint method, and verified the robustness of the comparison via uncertainty analysis. The variability of environmental impacts in different settings was explored using scenario analyses for the available minimum to maximum ranges.

**Results:**

Compared to a single physical visit in the studied setting, a telemedicine visit contributed less to global warming (0.1 vs 0.3 kg of carbon dioxide equivalents [kgCO_2_eq]; –60%), particulate matter formation (6.2 * 10^–9^ vs 1.8 * 10^–8^ disease incidence; –60%), and fossil resource use (1.8 vs 4.4 megajoules; –60%). Mineral/metal resource use was higher for telemedicine than for physical visits (1.1 * 10^–5^ vs 4.0 * 10^–6^ kg antimony equivalent; +180%). Only water use was not consistently different in the uncertainty analysis. Scenario analyses indicated that telemedicine’s environmental impact could become similar to physical visits only in urban settings (1-3 km of travel distance) with 50%-100% car commuting (0.1-0.4 vs 0.2-0.7 kgCO_2_eq). In rural settings (5-15 km of travel distance, 80%-100% car commute), physical visits’ environmental impact was higher (1.0-3.5 kgCO_2_eq), mostly even for mineral/metal resource use.

**Conclusions:**

Using telemedicine for domiciliary care and home nursing mostly reduces its environmental impact compared to physical visits. Benefits are larger in rural settings, where travel distances between patients are larger, and apply to multiple environmental impacts but not always to mineral/metal resource use. In urban settings, factors that influence the degree to which telemedicine is environmentally beneficial are whether staff are working from home versus at the office, commuting to the office by bicycle versus by car, and reusing video-calling devices. Accordingly, considerate application of telemedicine is important to support care for both human and planetary health.

## Introduction

The health care sector contributes notably to negative environmental impacts, accounting for up to 5% of global greenhouse gas emissions and air pollution [1]. National impacts can be higher, such as in the Netherlands, where the health care sector contributes 7% of greenhouse gas emissions and 13% of resource use [2]. In the face of adverse consequences for human and ecosystem health [3,4], there is an urgent need for health care to play its part by operating within planetary boundaries [5,6]. Therefore, countries increasingly set ambitions to transition to environmentally sustainable health care, focusing on, for example, resource use, energy consumption, and patient travel [7,8].

Telemedicine is often considered to be a promising solution for sustainable health care delivery as multiple reviews reported a reduction in travel-related emissions [9,10]. Savings were typically setting dependent and ranged anywhere between 0.7 and 372 kg of carbon dioxide equivalents (kgCO_2_eq) per consultation [10]. However, the underlying environmental impact assessments mostly did not follow international and transparent reporting standards, did not include outcomes other than greenhouse gas emissions, and failed to analyze impacts associated with telemedicine equipment or staff travel [11-13]. Moreover, earlier assessments took place in clinical contexts, leaving a knowledge gap for care delivery outside of resource-intensive hospital buildings [14], such as domiciliary care and home nursing (or nursing care at home [NCH]), wherein patient travel is minor or absent.

This knowledge gap is particularly relevant, as telemedicine in primary care and nursing has taken flight over recent years, especially during the COVID-19 crisis [15,16]. Telemedicine is suggested to benefit care access, patient outcomes, and nursing staff shortages—although evidence remains equivocal [17,18]. In the Netherlands, video calling for NCH and informal caretaker support have especially gained popularity. Therefore, the aim of this study was to quantify and compare the environmental impact of physical visits and telemedicine visits in the context of NCH.

## Methods

### Study Design

We conducted a comparative life cycle assessment (LCA) of NCH, following international standards regarding the conduct and interpretation of LCAs (ISO-14040/44) and a transparency checklist for quantifying greenhouse gas emissions of telemedicine (Supplement S1 in Multimedia Appendix 1) [12,19]. Between March 2023 and January 2024, we collected data for NCH visits, either as in-person visits or using video calling via a tablet. We collaborated with a service company (Compaan, the Netherlands) contracted by multiple NCH organizations in the Netherlands to provide tablets, telemedicine software, and a dedicated server. The company provided us with anonymous user data and connected us with NCH organizations using their service.

### Ethical Considerations

Ethical approval was waived by the authorized hospital review committee (file number 24-033). Contact persons of NCH organizations were informed regarding the purpose of the study and gave verbal consent upon first contact via phone or email. Their data was registered anonymously. Considering that telemedicine user data as registered by the telemedicine service company is per definition anonymous, no informed consent was obtained. There was no compensation for participation in the study.

### Care at Home

We contacted four NCH organizations in various urban areas in the Netherlands (Sensire, Zonnehuisgroep Amstelland, Careyn, and Pro Cura), providing domiciliary care and home nursing. Each organization predominantly served an older population with diverse health problems. Most patients received two “visits” per day, of which one required a physical presence (eg, wound dressing) and the other could be delivered via telemedicine, such as supporting patients in taking their medication or in structuring their daily agenda. Every patient using the telemedicine service had a dedicated tablet, including a user-friendly case and software design, for video calling with the NCH organization and for other applications such as an agenda and games. Once patients no longer used the service, tablets were returned for reuse. Based on the 1-3 years of experience of the NCH organizations, we considered physical visits and telemedicine visits to offer the same quality of care for patient support that did not require a physical visit. Patients and staff were generally positive about the introduction and use of telemedicine. Patients’ ability to use the service was verified in regular preceding physical visits.

### Data Collection

**Figure 1:**
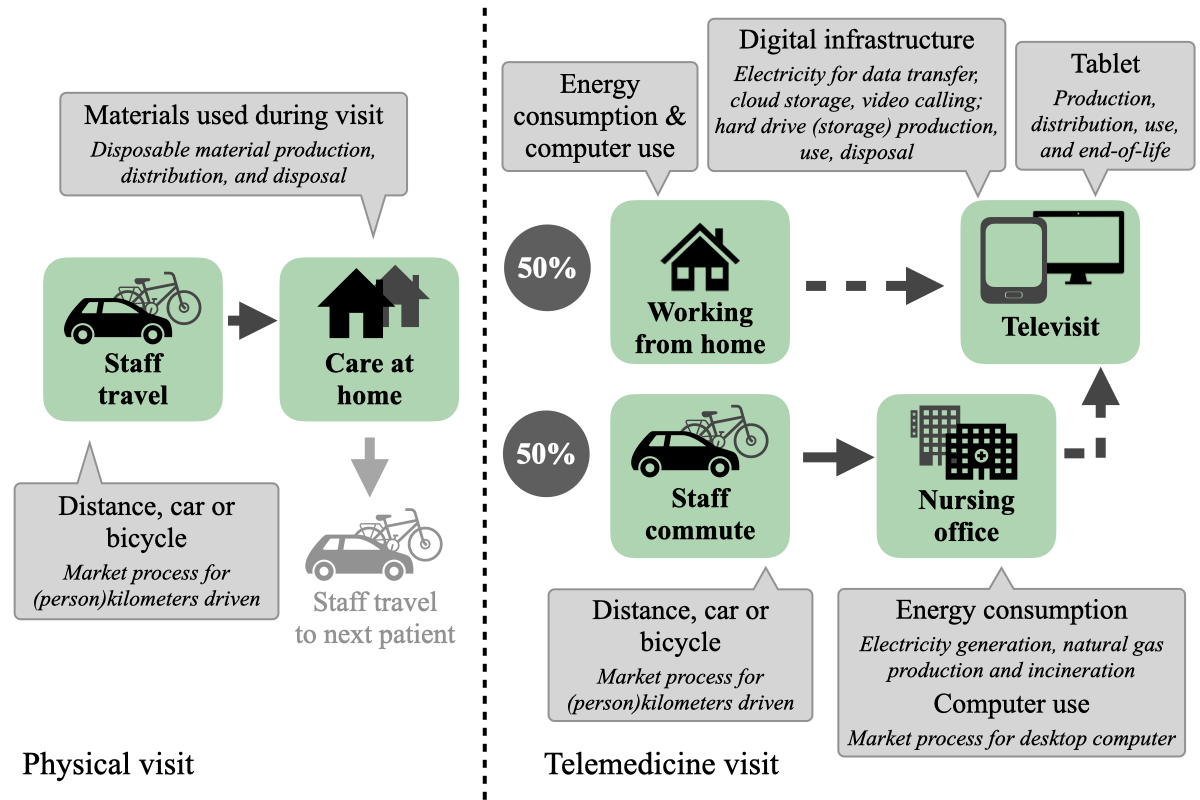
System boundaries of the life cycle assessment for nursing care at home.

We collected data for all resources required per individual patient visit (the “functional unit”), from resource extraction to use or disposal (“cradle to grave”). Where relevant for the type of visits, we included the following elements in the comparison: telemedicine tablets, staff commute, office building energy and computer use, materials used during physical visits, and the digital infrastructure required for the telemedicine service (Figure 1). Data were modeled in SimaPro LCA software v9.5.0.1 (PRé Sustainability). We combined self-collected data with generic background information on other life cycle stages such as the production of plastics, derived from the Ecoinvent v3.9 database [20]. For vehicle use and electricity generation, we used tailored data to accurately represent current standards in the Netherlands [21,22]. Since no relevant data were available from NCH organizations, we consulted two staff members per organization to shape assumptions regarding averages and minimum to maximum ranges for data, such as staff travel, staff commute, and duration of visits. A detailed overview of the life cycle inventory, the data collection, and the underlying assumptions is available in Supplement S2 in Multimedia Appendix 1.

Figure 1 provides an overview of the elements included in the scope of the analysis (green). The left side of the figure depicts an in-person visit, the right side a telemedicine visit, wherein the nursing staff can either be working from home or at the office (assumed 50% of the time).

### Study Outcomes

We used the Environmental Footprint method (v3.1), a methodology developed by the European Commission to quantify the environmental impact of products or services, intended to harmonize impact assessments in Europe [23]. Accordingly, we reported the categories of global warming in kgCO_2_eq, particulate matter formation in cumulative change in disease incidence per kg of PM_2.5_ or precursors (disease incidence), fossil resource use in megajoules (MJ), mineral/metal resource use in kg antimony equivalent (kgSbeq), and water use in m^3^.

### Data Analysis

We calculated the environmental impacts of physical visits for mean values (“reference scenario”) and reported the differences with telemedicine visits as a percentage of these environmental impacts. The impact of tablet production was allocated based on the total number of visits per patient that they were used for. To verify the robustness of the findings, we performed sensitivity analyses to test the effects of underlying database choices in the LCA model. Monte Carlo simulations of 1000 runs served as an uncertainty analysis for the investigated reference scenarios, using reported ranges of collected data and pedigree matrix-computed ranges of background data [24]. To explore how environmental impacts could vary in different settings, we used scenario analyses (“impact scenarios”) for available minimum to maximum ranges of telemedicine visits per nurse, staff commute, and staff travel between physical visits.

## Results

### Description of the Visits

NCH staff traveled an average of 1.5 (range 1.0-3.0) km between patients, of which an estimated 80% was by car and 20% by bicycle. Staff conducted telemedicine visits partially from home (assumed 50%) and had an average work-home commute of 12 (range 4-20) km. They conducted an average of 30 (range 20-40) telemedicine visits per day, lasting 3-10 minutes each and serving approximately 150 patients in total. On average, each tablet had been used by two patients consecutively for 278 (range 193-414) days each. Details are reported in Supplement S2 in Multimedia Appendix 1.

### Environmental Impact

**Figure 2:**
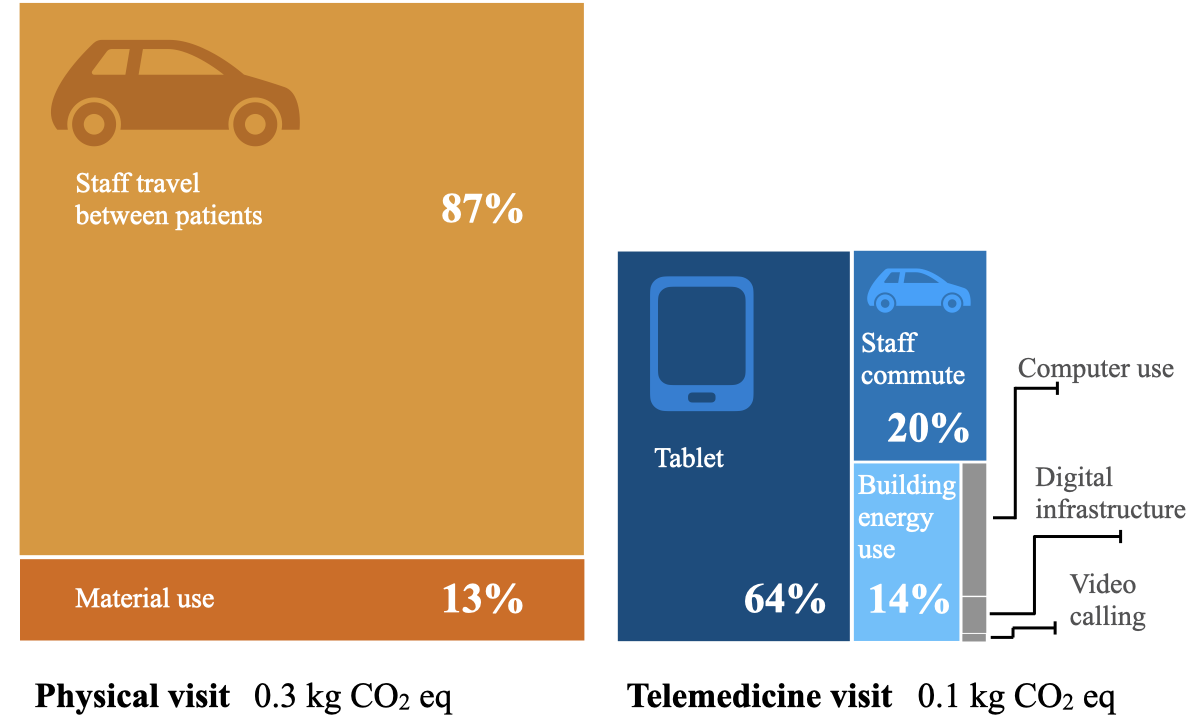
Contribution analysis of physical visits’ and telemedicine visits’ contribution to global warming. kg CO2 eq: kg of carbon dioxide equivalents.

Compared to a single physical visit in the reference scenario, a telemedicine visit contributed less to global warming (0.1 vs 0.3 kgCO_2_eq; –60%). Telemedicine also had a lower environmental impact regarding particulate matter formation (6.2 * 10^–9^ vs 1.8 * 10^–8^ disease incidence; –60%), fossil resource use (1.8 vs 4.4 MJ; –60%), and water use (6.2 * 10^–2^ vs 9.6 * 10^–2^ m^3^; –40%). For mineral/metal resource use, telemedicine contributed more than physical visits (1.1 * 10^–5^ vs 4.0 * 10^–6^ kgSbeq; +180%). The main contributors to the environmental impact of telemedicine visits were the production and use of the tablets (64% of total impact), followed by staff commute to the office (17%) and office building energy use (14%; Figure 2). The main contributor in physical visits was staff travel between patients (78%). Details are reported in Supplement S3 in Multimedia Appendix 1.

Figure 2 provides a comparative overview of the individual contributions of different elements to the total global warming caused by physical visits (orange, left) and telemedicine (blue, right). Values ≤2% are not indicated. The relative difference in surface area of both treemaps represents the difference in environmental impact for the reference scenarios (average values).

### Sensitivity and Uncertainty Analysis

Database choices for vehicle use (≤0.2 kgCO_2_eq), electricity generation (<0.1 kgCO_2_eq), and power use effectiveness for data transfer (<0.1 kgCO_2_eq) did not alter the results of the comparison. In the uncertainty analysis of the reference scenarios, telemedicine had a significantly lower contribution (>95% of runs) to global warming, particulate matter formation, and fossil resource use, and a higher contribution (95% of runs) to mineral/metal resource (Supplement S3 in Multimedia Appendix 1). Water use was not consistently different for both types of visits.

### Impact Scenarios

**Figure 3:**
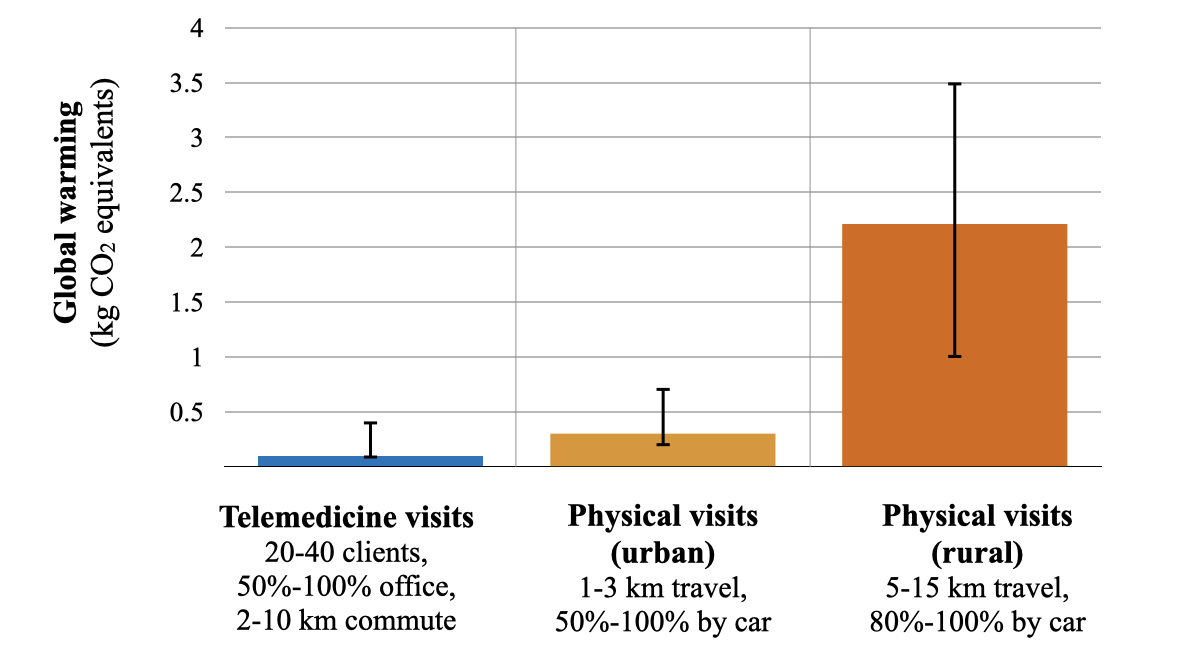
Telemedicine visits’ and physical visits’ contribution to global warming for different impact scenarios.

**Figure 4:**
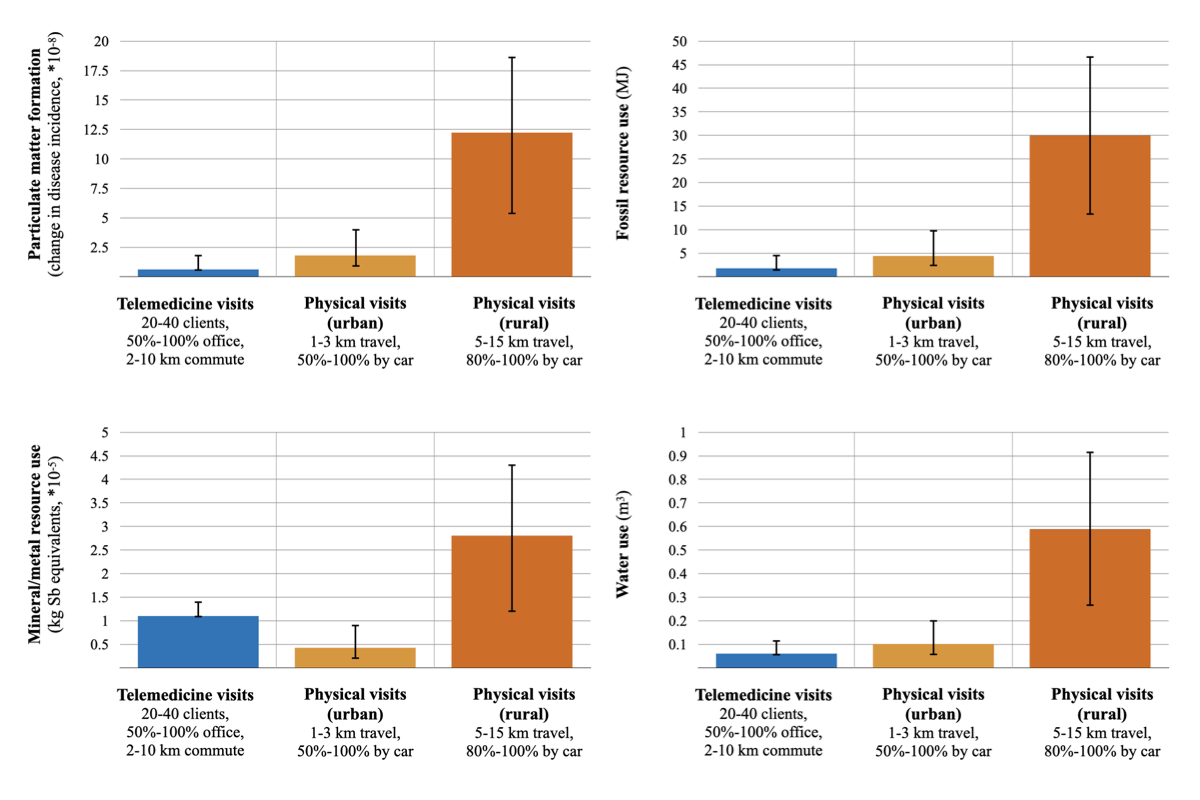
Telemedicine visits’ and physical visits’ contribution to particulate matter formation, fossil resource use, mineral/metal resource use, and water use for different impact scenarios.

Based on minimum-maximum ranges, telemedicine’s contribution to global warming could range between 0.1-0.4 kgCO_2_eq (Figure 3; Supplement S3 in Multimedia Appendix 1), including a worst-case scenario wherein 100% of staff commuted to the office by car for ≥10 km and each conducted the lowest number of visits (n=20) per day. Physical visits’ contribution to global warming could range between 0.2-0.7 kgCO_2_eq in a best-case scenario of short (1 km) staff travel between patients, partially by bicycle (50%). In more rural settings (5-15 km between patients), physical visits’ contribution to global warming could range between 1.0-3.5 kgCO_2_eq. Environmental impact categories other than global warming showed similar results (Figures 4; Supplement S3 in Multimedia Appendix 1), including the possibility of higher mineral/metal resource use for physical visits in rural settings.

Figures 3 and 4 provide a comparison of possible scenarios for telemedicine visits (blue), physical visits in an urban setting (orange), and physical visits in a rural setting (dark orange). The colored bars denote the reference scenarios reported for telemedicine visits and physical visits in an urban setting; for the rural setting, the average value was computed. The error bars indicate the range between the possible minimum and maximum values, based on the variables listed underneath the column. Note that these represent an exploration of possible scenarios for telemedicine and physical visits (ie, not the likelihood of values nor CIs). Details regarding the underlying scenarios are reported in Supplement S3 in Multimedia Appendix 1.

## Discussion

### Main Findings

In the studied NCH setting, telemedicine visits had a smaller contribution to global warming, particulate matter formation, and fossil resource use than physical visits. Mineral/metal resource use was larger for telemedicine than for physical visits. Only water use was not consistently different in the uncertainty analysis. Scenario analyses indicated that telemedicine’s environmental benefits were amplified if telemedicine was applied in settings with larger travel distances between patients, also resulting in equal or higher mineral/metal resource use associated with staff travel. In settings with short travel distances, staff commutes to the office influenced whether telemedicine was more or less favorable than physical visits.

### Contextualization

In recent years, multiple reviews reported lower greenhouse gas emissions for telemedicine [9,13,14]. Emission reductions paralleled the avoided travel distances, with an IQR of 52-386 km round trip per consultation in the most recent review [11]. Despite the much shorter travel distances in our study (1-15 km between patients), we also found travel to be the dominant source of carbon emissions in the studied setting. While absolute differences between telemedicine and physical visits were small per individual patient, environmental benefits should be considered cumulatively for the annual total of daily visits. Moreover, when applied in more rural settings or less densely populated countries with similar NCH services, telemedicine-associated environmental benefits will be larger. To date, no other study has investigated the environmental impact of telemedicine for nursing or NCH.

By including more elements of care in the scope of this LCA, we additionally demonstrated the impact of staff commuting to the office and telemedicine device use. Whereas the size of these impacts will vary based on the setting and way that telemedicine is applied, our findings do corroborate previous calls to consider device use and staff commute when studying the environmental impact of telemedicine [9,11]. Furthermore, preceding telemedicine studies rarely reported impacts other than global warming, leaving other negative consequences of health care’s environmental impact unaddressed [9,11]. Our study targets these research gaps and demonstrates the importance of a transparent and comprehensive assessment following international standards—including impacts other than CO_2_ emissions—to consider the potential shifting of environmental burdens and mineral/metal resource use associated with telemedicine devices.

### Limitations

Due to the unavailability of staff- and patient-related data from NCH organizations, we needed to make assumptions regarding means of travel, distances between patients, and frequency and duration of device use based on consultation with NCH staff members. As these assumptions may influence the LCA results, we accordingly conducted an uncertainty analysis and explored minimum-maximum ranges in different impact scenarios to account for variability in different settings. Moreover, to facilitate potential adjustments, we transparently shared our LCA model and calculations, and used a sensitivity analysis to quantify the effect of our database choices.

### Implications

First, we argue that the reported environmental benefit of telemedicine favors its rapid implementation in NCH, especially in settings or countries where travel distances are larger. Reuse of devices among consecutive patients and only distributing devices to patients who do not have their own can be additional strategies to limit the associated mineral/metal resource use [25]. Once more, it merits emphasis that replaced visits were elementary in nature, and the NCH organizations verified patients’ ability to safely use the service. Considering that implications for patient-related outcomes remain largely unstudied [26], the use of telemedicine for more complex nursing care should be practiced with due caution. Furthermore, while some patients may enjoy more privacy using a tablet, others may miss the physical contact. We therefore encourage future implementation studies to consider multiple domains of health care quality and ethics (including the environmental impact), as suggested by previous researchers [9].

Second, we speculate that telemedicine has additional benefits: it could enable some staff with physical health problems to conduct visits virtually, which they would otherwise be unable to conduct. Considering a growing demand for NCH in aging populations [27], alleviating nursing staff shortages and increasing efficiency would be another strong argument for the implementation of telemedicine. However, our study was not equipped to scientifically verify these experiences in participating NCH organizations, nor has such an effect been studied extensively for other digital technologies in nursing [26]. While future research may yield more evidence-based conclusions, continued education regarding digital competencies is important to equip nursing professionals to work with technologies such as telemedicine and can strengthen the potential benefits of its implementation [28].

### Conclusions

Using telemedicine for NCH mostly reduces its environmental impact compared to physical visits. Benefits are larger in rural settings, where travel distances between patients are larger, and apply to multiple environmental impacts, global warming, particulate matter formation, and fossil resource use, but not always to mineral/metal resource use. In urban settings, factors that influence the degree to which telemedicine is environmentally beneficial are whether staff are working from home versus at the office, commuting to the office by bicycle versus by car, and reusing video-calling devices. Accordingly, considerate application of telemedicine is important to support care for both human and planetary health.

## Appendix

Multimedia Appendix 1 Transparency checklist for quantifying greenhouse gas emissions of telemedicine, detailed overview of life cycle inventory and modeling, and life cycle impact assessment and sensitivity/uncertainty analysis.

## Abbreviations

kgCO2eq: kg of carbon dioxide equivalents
kgSbeq: kg antimony equivalent
LCA: life cycle assessment
MJ: megajoule
NCH: nursing care at home

## Data Availability

As much data as possible related to the life cycle inventory and modeling choices has been made available in the manuscript and Multimedia Appendix 1 to allow for a more detailed appreciation of the environmental impact assessment, quality control, and further use of the study findings. Considering the substantial amount of time required to export processes from SimaPro in the Excel format and convert them to reader-friendly Word files, the additional files can be obtained from the authors upon reasonable request within a reasonable time frame. Moreover, all processes will be uploaded to the freely accessible HealthcareLCA database in due time.
