# Supplementary material for: Environmental Impact of Physical Visits and Telemedicine in Nursing Care at Home: Comparative Life Cycle Assessment
Source: J Med Internet Res. 2025 Apr 4;27:e67538. doi: 10.2196/67538 (PMC12008697; doi:10.2196/67538)
Supplement: Multimedia Appendix 1 [file jmir_v27i1e67538_app1.pdf]

Supplementary material to

The environmental impact of physical visits and telemedicine in nursing care at home: a comparative life cycle assessment

Table of contents

Supplement S1 – Transparency checklist for quantifying greenhouse gas emissions of telemedicine — page 2

Supplement S2 – Detailed overview of life cycle inventory and modeling — page 3–6

Supplement S3 – Life cycle impact assessment and sensitivity/uncertainty analysis — page 7–15

References — page 16

Supplement S1 – Transparency checklist for quantifying greenhouse gas emissions of telemedicine

Item	Criterion	Relevant information
1	Does the study specify its aim?	Introduction section, last paragraph
2	Does the study specify its functional unit?	Methods section, ‘data collection’
3	Does the study specify its reference flow?	Specified in methods section, Figure 1, and Supplement B
4	Does the study provide a description of the life cycle stages?	Where applicable, life cycle stages are mentioned in the methods section, Figure 1, and Supplement B
5	Does the study provide a list of important unit processes?	Information regarding included processes provided in Supplement B, statement regarding availability at the bottom of Supplement B
6	Does the study specify exclusions and reasons for exclusions?	The study was based on anonymous user data, no exclusions (or inclusions) applied.
7	Does the study specify the system boundary?	Specified in methods section, Figure 1, and Supplement B
8	Does the study provide the source for all data used in the analysis?	Specified in Supplement B
9	Does the study assess the temporal representativeness of the data?	Reference years of used data are included in Supplement B. Based on availability, the most recent data were used to optimize temporal representativeness (e.g. for Power Use Effectiveness (PUE) of data transfer). Representativeness was included in the sensitivity analysis for the reference scenario by using pedigree matrix-computed uncertainty ranges.
10	Does the study assess the geographical representativeness of the data?	Where possible, the most geographically representative data were used. This relates most to the sources of energy generation and types of vehicles used – for which we used datasets tailored to the Netherlands. Representativeness was included in the sensitivity analysis for the reference scenario by using pedigree matrix-computed uncertainty ranges.
11	Does the study assess the technological representativeness of the data?	Where possible, the most technologically representative data were used. This relates most to the tablet, the PUE, and the production of SSD storage. Representativeness was included in the sensitivity analysis for the reference scenario by using pedigree matrix-computed uncertainty ranges.
12	Does the study assess the completeness of the data?	Statements regarding completeness of foreground data (discussion, limitations section) and background data (Supplement B) were included. During the research, EvB and LS assessed the completeness of the dataset at several timepoints and added information (e.g. the direct emissions due to combustion of natural gas) when available.
13	Does the study estimate the carbon footprint in terms of CO2eq?	Methods section, ‘study outcomes’
14	Does the study provide a list of greenhouse gasses taken into account?	Where information is available, the datasets in the study contain information regarding multiple greenhouse gases (carbon dioxide, methane, nitrous oxide, PCFs, HCFs, and sulphur hexafluoride)
15	Does the study specify the selected characterization factors?	Methods section, ‘study outcomes’. Characterization factors are included in the Environmental Footprint method v3.1 (based on scientific consensus). Kindly refer to the guiding documents of the Joint Research Centre (JRC) of the European Commission for further information.
16	Does the study report the selected allocation procedures?	Methods section, ‘data analysis’, and Supplement B
17	Does the study report the outcomes per unit of analysis?	The results were reported according to the functional unit
18	Does the study report the carbon footprint separately per specific component?	Results section, ‘environmental impact’, and Supplement C
19	Does the study report the carbon footprint according to life cycle phases?	We reported environmental impacts based on elements of care included in the reference flow. We included a statement regarding the life cycle contribution of the tablets in the Results section, ‘environmental impact’
20	Does the study report a qualitative statement on the influence of key uncertainties or methodological choices on the result?	Results section, ‘sensitivity and uncertainty analysis; Discussion section; and Supplement C
21	Does the study perform a quantitative sensitivity analysis?	Results section, ‘sensitivity and uncertainy analysis’ and ‘impact scenarios’; Supplement C
22	Does the study critically discuss limitations, e.g., appropriateness of system boundary, data quality, or methods of analysis?	Limitations regarding foreground data are discussed in the Discussion section, ‘limitations’ and ‘contextualization’; more detailed statements regarding specific life cycle inventory elements are included in Supplement B (e.g. data transfer / storage / computing)

Note to reader: this table of assessment criteria is based on Table 1 of the article *A Transparency Checklist for Carbon Footprint Calculations Applied within a Systematic Review of Virtual Care Interventions* by Lange et al (2022), doi: 10.3390/ijerph19127474

Supplement S2 – Life cycle inventory and modelling

Table S2.1. Life cycle inventory overview

Process category	Subcategory	Data collection	Data modelling LCI
Tablet	Tablet	Information regarding the type of tablet used (mid-range Samsung tablet: A8 or A2019), the average number of users per tablet over the course of its device life (2-3) and the average number of calls (278) per patient were obtained from the telemedicine service company and their anonymous user data.<br><br>As an alternative (= "proxy"), we used the publicly available information for an Apple iPad 2017.	Own modelling using ecoinvent market processes, based on several publicly available sources: mainly Arduin et al (2017)[1], but adjusted and expanded to more accurately reflect the process of modern handheld/tablet devices (including production and packaging using the detailed LCA appendices of the Fairphone by Merve Güvendik (2014)[2].<br><br>To investigate differences, we compared the manufacturer LCA data with our own environmental impact calculations for the proxy tablet.
Commute	Staff travel	Average means of travel and travel distance based on information of three medium-large nursing home care organizations (2024) and interviews with healthcare professionals of nursing home care organizations (2023). We estimated that 80% of employees travel by car for a distance of 1-3 km.<br><br>Since travel distances between patients vary based on the region where the nursing care at home takes place, we chose to explore different travel distances (especially for more rural settings) in different scenario analysis.	The transportation processes tailored to the Dutch market for transportation of goods (Goederenvervoer STREAM 2020; CE Delft, 2021)[3] and commute of persons (Personenvervoer STREAM 2022; CE Delft, 2023)[4] were used as market processes (S/U), adjusted from the standard cut-off by classification market processes in ecoinvent v3.9.1 for the same fuel types and vehicle types. The adjusted processes were previously commissioned by the Dutch Government. Adjustments are made for the processes that represent the production of fuels, such as gasoline (i.e. Well-To-Tank emissions), e.g. gasoline currently contains 10% biofuels, rather than 5% biofuels, resulting in a lower requirement of crude oil and higher requirement of land use for production of biofuels. And adjustments are made for the processes that represent the combustion of fuels and driving of vehicles (i.e. Tank-To-Wheel emissions), e.g. the amount of PM2.5 that is emitted during the process of driving a car, based on direct measurements of Dutch vehicles.<br><br>Effectively, this results in: 1) replacement of the CO2eq, NOx, SO2, NMVOC, and PM2.5 emissions documented in ecoinvent processes by the vehicle measurements conducted in the Netherlands; 2) for commute of persons the reporting unit of cars and motorbikes was adjusted based on the average occupation of vehicles ('km' to 'personkm'), such as a factor 1.31 for the average car. For further details/reporting, kindly consult the authors.<br><br>Means of travel and travel distance were modelled in a process representing the 'average' individual staff member for one patient visit (even though in real life an individual

			cannot be travelling by car and bicycle at the same time). Average percentages for means of transport (e.g. 80% car transport) were used to allocate 80% of the travel distance by car and the remaining 20% by bicycle or public transport.
	Staff commute	Average distance home to work based on contact with convenience sample of three nursing organizations (employees live 2-10km away from the office). Distances were double counted to represent a return journey.<br><br>Means of transport assumed to be 50% travel by car, 30% by bike, 10% by e-bike, and 10% by public transport (either bus or train; both assumed to be 50%).<br><br>Number of patients seen per day (20-40) and percentage of employees working from home (50%) based on contact with two nursing organizations and experience of telemedicine service company.	Explanation of transportation process as explained above for "staff travel"<br><br>Own modelling of commute distance between home and the office for the member of staff conducting the telemedicine nursing visit. Allocation of commute based on allocation of staff workload to the care for a single patient AND staff working from home (e.g. impact of travel was reduced by 50% to discount the employees working from home and divided by the number of patients seen per day for allocation).<br><br>Modelled travel distance in a process representing the 'average' nursing home care employee. Means of travel (e.g. 50% car travel) were used to allocate 50% of the travel distance by car and the remaining percentage by bicycle or public transport.
Building energy use	Building energy use	Average energy use for nursing office building, based on a recent reference dataset for energy use in Dutch office buildings “kengetallen Milieubarometer kantoor” (2022, Stimular) [5]. This includes 63 kWh/m2/year for electricity and 2.7m3eq/year for natural gas consumption.<br><br>Surface area allocated to a single employee (for their working day) was assumed to be 10-16 m2, based on descriptions of office environment in conversations with nursing staff and telemedicine service provider.<br><br>It was assumed that -when working from home – no additional energy use occurs inside the staff member’s home, except for the energy consumption of the computer used for telemedicine, which is included in the input “computer use” below.	The electricity mix tailored to the Dutch market in 2021 "Elektriciteit gemiddeld - NL 2021" is a market process (S) that was created by CE Delft (Delft, the Netherlands) in 2023-2024 [6], commissioned by the Dutch Government and based on energy generation data as collected by the Dutch National Institute for Statistics (CBS) and National Planning Bureau for the Environment (PBL).<br><br>Converted to energy use per day, allocated to visit of a single patient based on (assumed) size of nursing office workspace. Further allocated to individual patient based on average workload (no. of patients and admin work performed by the nursing staff) and duration of the telemedicine visit. Separately added incineration of natural gas. Similar to the staff commute, 50% of the environmental impact was subtracted to account for nursing staff working from home (and therefore not using the office building).
Materials used during visit	Hand disinfectant	Usage based on own assumption, derived from clinical experience.	Own modelling using ecoinvent market processes. Relevant products based on previous publication (Thiel et al 2023)[7] and previous research in other Dutch academic hospital.
	Nitrile glove	Usage based on own assumption that nurse may use gloves in 25% of the visits. Confirmation that gloves are not standard use during visits confirmed in conversations with nursing staff.	Own modelling using ecoinvent market processes. Materials and processes, including energy use for air leak testing, based on Jamal et al (2021)[8].
	Face mask	Usage based on own assumption that nurse may use one face mask over the course of visiting 15 patients.	Own modelling using ecoinvent market processes. Materials and processes based on Rizan (2021)[9].
Digital infrastructure	Data transfer / storage / computing	Required number of storage sites, location of storage, and number of data transfers based on interviews with	Explanation of electricity mix as stated in "Building energy use".

		telemedicine service provider employees and personal email communication with IT-specialists of hosting server company. Required amount of energy for data transfer and cloud storage based on consultation of knowledge management/IT experts and existing publications with a recent overview of Power Use Effectiveness (PUEs) (Jackson et al 2023, Swiss Federal Office of Energy 2022). [10,11]<br><br>Required amount of data (to store application-associated information) based on own assumption (100MB–1GB) and conversation with telemedicine service company.<br><br>Information regarding production of SSD storage based on publicly available LCA information of SSD-manufacturer.	Modelled data transfer and online storage for assumed data size (in GB), including the 'Jackson' and 'Swiss' process for the PUEs where possible and comparing them in sensitivity analysis. Based on geolocation of online storage ("mirror sites"), chose the German or French electricity mix as available in ecoinvent accordingly. Due to limited data availability, only the electricity use is included in the model. Water use for cooling is not included.<br><br>Environmental impact of SSD production (based on required data storage size, in GB) was entered directly as environmental impact based on Seagate (2016) [12] LCA reporting. Added Tannu et al (2022) [13] review for recent comparison - only available for carbon footprint of SSD production.
	Computer use (by staff)	Duration of usage (10-15 min per patient) based on contact with two nursing organizations and experience of telemedicine service company.	Own modelling using adjusted ecoinvent market process. Excluded energy use of computer by 50% to avoid double counting when employees are working from the office (since energy use is already included in the Building Energy Use process).
	Video call	Duration based on average duration for calls via the telemedicine service provider platform (= anonymous user data).<br><br>Assumed requirement of approx 225-675MB (450MB) for video call per hour (gadgetstouse.com/businesstechplanet.com).	Explanation of electricity mix as stated in "Building energy use". Explanation of PUE modelling for data transfer (required energy to transfer average data size) as stated in "Data transfer / storage / computing".<br><br>Input modelled for duration of 15 minutes and conversion MB to GB (/1000).

NB: for all products, plastic- and metal specific incineration processes were modelled for their end-of-life; if specific plastics were unavailable or if disposables consisted of other materials, the municipal incineration was chosen, except for minor parts of the tablet, which were included as 'electronic waste' (process available in ecoinvent).

Table S2.2. Modelling of comparison Physical Visit and Telemedicine

**SimaPro 9.5.0.1**	**LCA: Telemedicine**				
**Process**	**Amount**	**Distribution**	**Upper**	**Lower**	**Remarks**
Video call (15 min)	0.5	Triangle	0.3	0.8	Process accounting for energy required to transfer data during video call. Assumed 7,5 min video call between nursing home care organisation and client; triangular distribution 4,5 -12 minutes.
Computer use, per hour [Ecoinvent EU proxy 2005, CE Delft energy adjusted 2021]	0.2	Uniform	0.2	0.25	Process accounting for computer usage by nursing home care staff during video call, incl approx allocation of stand-by and off-time out of office hours. Used for 10-15 min per patient in total (incl pre and post call).
Staff commute	1	n/a	n/a	n/a	Process accounting for commute of employees to the office where telemedicine is done. Unit represents allocation to a single patient. Refer to process for details (includes 50% working from home allocation).
Building energy use	0.5	n/a	n/a	n/a	Process accounting for energy usage of workstation where remote care is delivered. Considering 50% of employees work from home, only 50% is allocated (no attributed energy/gas in home setting).
Tablet (iPad 2017 proxy)	Tablet_allocation	n/a	n/a	n/a	Allocation function for single visit is defined below.

Telemedicine digital infrastructure	1	n/a	n/a	n/a	Content of digital infrastructure is explained in Table A1.
**Based on:**					
**Input parameter**	**Amount**	**Distribution**	**Lower**	**Upper**	**Remarks**
Tablet_allocation	1*functional_allocation / number_of_calls / client_reuse	n/a	n/a	n/a	Calculated allocation factor for tablet, based on reuse, functional allocation (care delivery vs 'other benefits), and number of calls per client.
Functional_allocation	1	n/a	n/a	n/a	Based on allocation choice, only part of the tablet can be allocated to the actual care delivery and other parts to 'benefits' such as video calling with family members, playing games etc. Factor (multiplier) 1 = 100%. If 100%, all production of the tablet is allocated to the care delivery and 'benefits' are regarded to come at no extra environmental cost.
Number_of_calls	278	Uniform	235	414	Factor (divide) indicates the number of calls/moments of care delivery are executed using a single tablet for a single client. Sensing at two service locations (may 2023, Compaan data) indicates that tablets are used on average for 1 call per day. Average duration of tablets in use (general Compaan data) 2-3 years per client. Average with client: 278 days.
Client_reuse	2	n/a	n/a	n/a	Factor (divide) indicates how many times a single tablet is re-used by another client, before being disposed of. Based on Compaan data for multiple service organisations, the average tablet is used for 2 clients in total (although number is still increasing).

**SimaPro 9.5.0.1**	**LCA: Physical Visit**				
**Process**	**Amount**	**Distribution**	**Upper**	**Lower**	**Remarks**
Staff travel	1	N/a	n/a	n/a	Process accounting for transportation for a single visit. Average travel including car/(e-)bike. Refer to actual process for further details.
Hand disinfection	2	n/a	n/a	n/a	Assumed 2* hand disinfection: before and after visit to patient.
Face mask	1/15	n/a	n/a	n/a	Assumed that face mask is worn when visiting patients and that same face mask is used for 15 patient visits (assumption).
Nitrile glove	2*0,25	n/a	n/a	n/a	Assumed that 2 nitrile gloves are used 25% of the time (assumption).

Note to reader

Additional process data is available for each of the subcategories listed in Table B1, yet in Excel file format – and therefore not included in this supplement. Considering the substantial amount of time required to export processes from SimaPro in Excel format and converting them to reader-friendly Word files, the additional files can be obtained from the authors upon reasonable request within a reasonable timeframe. After publication of the study, all processes will be uploaded to the freely accessible HealthcareLCA database in due time.

Supplement S3 – Life cycle impact assessment and sensitivity/uncertainty analysis

Table S3.1. Life cycle impact assessment for Physical visits
Calculated using "Environmental Footprint 3.1 (adapted) V1.00 / EF 3.1 normalization and weighting set."

Damage category	Unit	Total	% of total	Transport - nursing home care average, patient visit	% of total	Hand disinfection	% of total	Nitrile glove	% of total	Face mask	% of total
Acidification	mol H+ eq	0,001262	100	0,00107295	85,0	0,00011471	9,1	6,90E-05	5,5	5,35E-06	0,4
Climate change	kg CO2 eq	0,31900129	100	0,27731446	86,9	0,02288877	7,2	0,01744498	5,5	0,00135309	0,4
Ecotoxicity, freshwater	CTUe	4,5076767	100	3,0297224	67,2	1,3874428	30,8	0,08665051	1,9	0,00386101	0,1
Particulate matter	disease inc.	1,75E-08	100	1,55E-08	88,7	1,29E-09	7,4	6,37E-10	3,6	5,15E-11	0,3
Eutrophication, marine	kg N eq	0,00034371	100	0,00030881	89,8	1,95E-05	5,7	1,42E-05	4,1	1,20E-06	0,3
Eutrophication, freshwater	kg P eq	5,59E-05	100	4,67E-05	83,7	5,28E-06	9,5	3,64E-06	6,5	1,93E-07	0,3
Eutrophication, terrestrial	mol N eq	0,00313403	100	0,00273049	87,1	0,00024309	7,8	0,00014775	4,7	1,27E-05	0,4
Human toxicity, cancer	CTUh	2,87E-10	100	2,74E-10	95,3	8,65E-12	3,0	4,44E-12	1,5	3,43E-13	0,1
Human toxicity, non-cancer	CTUh	4,12E-09	100	3,74E-09	90,8	2,56E-10	6,2	1,16E-10	2,8	7,85E-12	0,2
Ionising radiation	kBq U-235 eq	0,01079003	100	0,00880196	81,6	0,00117503	10,9	0,00076851	7,1	4,45E-05	0,4
Land use	Pt	2,3956822	100	2,2081813	92,2	0,07972046	3,3	0,10037536	4,2	0,00740506	0,3
Ozone depletion	kg CFC11 eq	1,65E-08	100	7,11E-09	43,1	9,14E-09	55,5	2,09E-10	1,3	1,16E-11	0,1
Photochemical ozone formation	kg NMVOC eq	0,00139456	100	0,00125055	89,7	8,21E-05	5,9	5,76E-05	4,1	4,27E-06	0,3
Resource use, fossils	MJ	4,447339	100	3,7406042	84,1	0,4630228	10,4	0,22432678	5,0	0,01938513	0,4
Resource use, minerals and metals	kg Sb eq	3,96E-06	100	3,52E-06	88,7	3,53E-07	8,9	8,93E-08	2,3	4,09E-09	0,1
Water use	m3 depriv.	0,09644783	100	0,07240945	75,1	0,01967429	20,4	0,00418845	4,3	0,00017564	0,2

Table S3.2. Life cycle impact assessment for Telemedicine (using iPad 2017 proxy data)

Calculated using "Environmental Footprint 3.1 (adapted) V1.00 / EF 3.1 normalization and weighting set."

NB: considering that the Samsung tablet data were only available in an older environmental impact assessment format (CML baseline 2000), we chose to report the results with the proxy tablet. As far as environmental impact categories could be compared (e.g. climate change / global warming), results for the Samsung tablet and proxy tablet were similar (0.13 kg CO_2 eq vs 0.12 kg CO_2 eq respectively for a single visit). Results for the Samsung tablet are reported in Table B3.

Damage category	Unit	Total	% of total	Tablet (iPad 2017 proxy)	% of total	Staff commute	% of total	Energy use	% of total	Computer usage (staff)	% of total	Digital infrastructure	% of total	Video call	% of total
Acidification	mol H+ eq	0,00067461	100	0,00052011	77,1	9,92E-05	14,7	3,22E-05	4,8	2,15E-05	3,2	1,32E-06	0,2	3,52E-07	0,1
Climate change	kg CO2 eq	0,13121359	100	0,08396221	64,0	0,02555119	19,5	0,0176689	13,5	0,00307172	2,3	0,00080164	0,6	0,00015793	0,1
Ecotoxicity, freshwater	CTUe	1,3967716	100	1,0338141	74,0	0,27344251	19,6	0,01951117	1,4	0,05695097	4,1	0,0128407	0,9	0,00021222	0,0
Particulate matter	disease inc.	6,23E-09	100	4,42E-09	70,9	1,43E-09	23,0	1,60E-10	2,6	1,75E-10	2,8	4,75E-11	0,8	1,71E-12	0,0
Eutrophication, marine	kg N eq	0,0001693	100	0,00012623	74,6	2,84E-05	16,8	9,05E-06	5,3	5,19E-06	3,1	3,22E-07	0,2	9,82E-08	0,1
Eutrophication, freshwater	kg P eq	6,70E-05	100	5,90E-05	88,1	4,23E-06	6,3	3,87E-07	0,6	2,77E-06	4,1	5,67E-07	0,8	3,98E-09	0,0
Eutrophication, terrestrial	mol N eq	0,00149392	100	0,00108804	72,8	0,00025481	17,1	0,0001025	6,9	4,49E-05	3,0	2,50E-06	0,2	1,11E-06	0,1
Human toxicity, cancer	CTUh	9,12E-11	100	5,82E-11	63,7	2,61E-11	28,6	2,90E-12	3,2	3,71E-12	4,1	3,61E-13	0,4	2,89E-14	0,0
Human toxicity, non-cancer	CTUh	6,47E-09	100	5,75E-09	88,9	3,37E-10	5,2	8,07E-11	1,2	2,94E-10	4,5	7,88E-12	0,1	8,71E-13	0,0
Ionising radiation	kBq U-235 eq	0,01096725	100	0,00907655	82,8	0,00082542	7,5	0,00030714	2,8	0,00028063	2,6	0,0004741	4,3	3,41E-06	0,0
Land use	Pt	0,58414058	100	0,32690589	56,0	0,20404512	34,9	0,03465682	5,9	0,01657075	2,8	0,00157901	0,3	0,000383	0,1
Ozone depletion	kg CFC11 eq	3,71E-09	100	2,25E-09	60,6	6,46E-10	17,4	6,76E-10	18,2	1,28E-10	3,4	5,84E-12	0,2	5,12E-12	0,1
Photochemical ozone formation	kg NMVOC eq	0,0004693	100	0,00030347	64,7	0,00011525	24,6	3,52E-05	7,5	1,30E-05	2,8	2,05E-06	0,4	3,65E-07	0,1
Resource use, fossils	MJ	1,7587424	100	1,0611812	60,3	0,34540871	19,6	0,29351574	16,7	0,03889294	2,2	0,01711221	1,0	0,00263152	0,1
Resource use, minerals and metals	kg Sb eq	1,12E-05	100	1,02E-05	91,4	3,07E-07	2,7	2,71E-08	0,2	6,25E-07	5,6	5,92E-09	0,1	2,93E-10	0,0
Water use	m3 depriv.	0,06213963	100	0,00938205	15,1	0,00628197	10,1	0,00221946	3,6	-6,71E-05	-0,1	0,04429869	71,3	2,46E-05	0,0

Table S3.3. Life cycle impact assessment for Telemedicine (using Samsung tablet data)
Calculated using "CML baseline 2000 / Netherlands, 1997 in SimaPro."

NB: impacts of tablet use (Samsung LCA data) have been added manually based on their own reporting. Manual additions are marked blue and refer to table B4.

Impact category	Unit	Total	% of total	Tablet	% of total	Staff commute	% of total	Energy use	% of total	Computer usage	% of total	Digital infrastructure	% of total	Video call	% of total
Abiotic depletion	kg Sb eq	7,29E-04	100,0%	3,70E-04	50,7%	0,00017137	23,5%	0,0001584	21,7%	2,30E-05	3,1%	5,66E-06	0,8%	1,42E-06	0,2%
Acidification	kg SO2 eq	5,99E-04	100,0%	4,77E-04	79,6%	7,85E-05	13,1%	2,50E-05	4,2%	1,72E-05	2,9%	1,08E-06	0,2%	2,73E-07	0,0%
Eutrophication	kg PO4--- eq	3,09E-04	100,0%	2,64E-04	85,5%	2,68E-05	8,7%	4,57E-06	1,5%	1,14E-05	3,7%	1,89E-06	0,6%	4,86E-08	0,0%
Global warming (GWP100)	kg CO2 eq	1,16E-01	100,0%	7,00E-02	60,1%	0,02511262	21,6%	0,01741192	15,0%	0,00300634	2,6%	0,00079659	0,7%	0,00015574	0,1%
Ozone layer depletion (ODP)	kg CFC-11 eq	7,17E-09	100,0%	5,86E-09	81,7%	6,03E-10	8,4%	5,45E-10	7,6%	1,58E-10	2,2%	5,31E-12	0,1%	4,18E-12	0,1%
Human toxicity	kg 1,4-DB eq	3,62E-01	100,0%	3,02E-01	83,5%	0,04056876	11,2%	0,00450622	1,2%	0,013896	3,8%	0,00073942	0,2%	4,32E-05	0,0%
Fresh water aquatic ecotox.	kg 1,4-DB eq	2,91E-01	100,0%	2,63E-01	90,4%	0,01776681	6,1%	0,00011144	0,0%	0,00937099	3,2%	0,00056388	0,2%	4,55E-07	0,0%
Marine aquatic ecotoxicity	kg 1,4-DB eq	3,83E+02	100,0%	3,47E+02	90,7%	18,305912	4,8%	2,5036351	0,7%	14,020834	3,7%	0,69804146	0,2%	0,01965185	0,0%
Terrestrial ecotoxicity	kg 1,4-DB eq	9,70E-04	100,0%	4,80E-04	49,5%	0,00039971	41,2%	3,40E-05	3,5%	5,31E-05	5,5%	2,47E-06	0,3%	3,64E-07	0,0%
Photochemical oxidation	kg C2H4 eq	2,78E-05	100,0%	1,78E-05	63,9%	7,50E-06	27,0%	1,50E-06	5,4%	9,69E-07	3,5%	5,73E-08	0,2%	1,46E-08	0,1%

Table S3.4. Reference table for impact calculations of Samsung tablet
NB: Values have been adjusted for the 'use phase' of the tablet, which was 2 years in the manufacturer LCA data (and therefore ½ of the use phase impact has been subtracted)..

Impact category	Unit	Value	Calculated	Times used		
Abiotic depletion	kg Sb eq	2,06E-01	3,70E-04	**278**	average	235-414 days range for clients (of which average 1 call per day)
Acidification	kg SO2 eq	2,65E-01	4,77E-04	Redistributed		
Eutrophication	kg PO4--- eq	1,47E-01	2,64E-04	**2**	average	
Global warming (GWP100)	kg CO2 eq	3,89E+01	7,00E-02	Functional allocation		
Ozone layer depletion (ODP)	kg CFC-11 eq	3,26E-06	5,86E-09	**1**	(100%, only used for Compaan)	
Human toxicity	kg 1,4-DB eq	1,68E+02	3,02E-01			
Fresh water aquatic ecotox.	kg 1,4-DB eq	1,46E+02	2,63E-01	Calculation per consultation:		
Marine aquatic ecotoxicity	kg 1,4-DB eq	1,93E+05	3,47E+02	*value * functional allocation / average use / redistribution*		
Terrestrial ecotoxicity	kg 1,4-DB eq	2,67E-01	4,80E-04			
Photochemical oxidation	kg C2H4 eq	9,87E-03	1,78E-05			

Table S3.5. Environmental impact comparison of Physical Visit and Telemedicine

Comparison based on impact assessment for Physical Visits (Table B1) and using the iPad proxy tablet data (Table B2). Calculated using "Environmental Footprint 3.1 (adapted) V1.00 / EF 3.1 normalization and weighting set."

Damage category	Unit	Total Physical Visit	Total Telemedicine	Difference (Telemedicine = 'n' times smaller (<1) or larger (>1))
Acidification	mol H+ eq	$1.3 * 10^{-3}$	$6.7 * 10^{-4}$	0.5
Climate change	kg CO2 eq	0.3	0.1	0.4
Ecotoxicity, freshwater	CTUe	4.5	1.4	0.3
Particulate matter	disease inc.	$1.8 * 10^{-8}$	$6.2 * 10^{-9}$	0.4
Eutrophication, marine	kg N eq	$3.4 * 10^{-4}$	$1.7 * 10^{-4}$	0.5
Eutrophication, freshwater	kg P eq	$5.6 * 10^{-5}$	$6.7 * 10^{-5}$	1.2
Eutrophication, terrestrial	mol N eq	$3.1 * 10^{-3}$	$1.5 * 10^{-3}$	0.5
Human toxicity, cancer	CTUh	$2.9 * 10\text{-}^{10}$	$9.1 * 10^{-11}$	0.3
Human toxicity, non-cancer	CTUh	$4.1 * 10^{-9}$	$6.5 * 10^{-9}$	1.6
Ionising radiation	kBq U-235 eq	$1.1 * 10^{-2}$	$1.1 * 10^{-2}$	1.0
Land use	Pt	2.4	0.6	0.2
Ozone depletion	kg CFC11 eq	$1.6 * 10^{-8}$	$3.7 * 10^{-9}$	0.2
Photochemical ozone formation	kg NMVOC eq	$1.4 * 10^{-3}$	$4.7 * 10^{-4}$	0.3
Resource use, fossils	MJ	4.4	1.8	0.4
Resource use, minerals and metals	kg Sb eq	$4.0 * 10^{-6}$	$1.1 * 10^{-5}$	2.8
Water use	m3 depriv.	$9.6 * 10^{-2}$	$6.2 * 10^{-2}$	0.6

Table S3.6. Sensitivity analysis of Physical Visits for different staff travel distances and means of transport between individual patients

NB: the different scenarios also serve as an exploration of the use of telemedicine in rural vs urban settings. These are incorporated in the error bars of Figure 3 of the article in the main text.

NB2: percentages listed indicate the employees traveling by car, the remaining percentages travels by bicycle. For the longest rural distances no travel by bicycle is listed, since there is not enough time available to cover the distance between patients by bike (in the nursing home care planning).

		Urban	*Urban*	*Urban*	*Urban*	*Rural*	*Rural*	*Rural*
Damage category	Unit	1km one way, 50% car	1km one way 80% car	3km one way 80% car	3km one way 100% car	5km one way 80% car	5km one way 100% car	15km one way 100% car
Acidification	mol H+ eq	0,000672	0,00090435	0,00233496	0,00279967	0,00376556	0,00454008	0,01324214
Climate change	kg CO2 eq	0,16286507	0,22656314	0,59631575	0,72371189	0,96606837	1,1783953	3,4518121
Ecotoxicity, freshwater	CTUe	2,7708433	3,4977693	7,5373992	8,9912511	11,577029	14,000116	39,044438
Particulate matter	disease inc.	8,95E-09	1,23E-08	3,30E-08	3,98E-08	5,38E-08	6,50E-08	1,91E-07
Eutrophication, marine	kg N eq	0,00016978	0,00024078	0,00065252	0,00079451	0,00106426	0,00130091	0,00383292
Eutrophication, freshwater	kg P eq	3,06E-05	4,03E-05	0,00010258	0,00012197	0,00016489	0,0001972	0,00057334
Eutrophication, terrestrial	mol N eq	0,00160412	0,00222386	0,00586451	0,00710399	0,00950516	0,01157096	0,0339058
Human toxicity, cancer	CTUh	1,39E-10	1,96E-10	5,61E-10	6,75E-10	9,26E-10	1,12E-09	3,32E-09
Human toxicity, non-cancer	CTUh	2,07E-09	2,87E-09	7,85E-09	9,46E-09	1,28E-08	1,55E-08	4,58E-08
Ionising radiation	kBq U-235 eq	0,00592581	0,00785605	0,01959199	0,02345247	0,03132794	0,03776206	0,10931004
Land use	Pt	1,1476647	1,6596218	4,6038635	5,6277777	7,5481053	9,2546289	27,388885
Ozone depletion	kg CFC11 eq	1,24E-08	1,41E-08	2,36E-08	2,69E-08	3,31E-08	3,86E-08	9,71E-08
Photochemical ozone formation	kg NMVOC eq	0,00068732	0,00097771	0,00264511	0,00322589	0,00431251	0,00528048	0,0155534
Resource use, fossils	MJ	2,3356708	3,2004709	8,1879432	9,9175433	13,175416	16,058082	46,760778
Resource use, minerals and metals	kg Sb eq	2,01E-06	2,79E-06	7,48E-06	9,03E-06	1,22E-05	1,48E-05	4,34E-05
Water use	m3 depriv.	0,05525281	0,07231134	0,16885728	0,20297434	0,26540321	0,32226498	0,91871818

Table S3.7. Sensitivity analysis of Telemedicine for different staff commute distances and number of patients
NB: the "worst case scenario" for Telemedicine indicates an employee only seeing 20 patients per day and commuting to the office by car for a distance of 10 km (rather than working from home or commuting to the office by bicycle). These are incorporated in the error bar of Figure 3 in the main text.
NB2: the reference scenario wherein 50% of employees work from home and 50% come to the office is not included in this table.

Damage category	Unit	20 online clients, office, car, 2km one way	20 online clients, office, car, 10km one way	30 online clients, office, car, 2km one way	30 online clients, office, car, 10km one way	40 online clients, office, car, 2km one way	40 online clients, office, car, 10km one way
Acidification	mol H+ eq	0,00081035	0,00150652	0,00072015	0,00118426	0,00067505	0,00102313
Climate change	kg CO2 eq	0,18489626	0,36676961	0,15207125	0,27332015	0,13565875	0,22659542
Ecotoxicity, freshwater	CTUe	1,6611252	3,664671	1,4746519	2,8103491	1,3814152	2,3831881
Particulate matter	disease inc.	7,63E-09	1,77E-08	6,63E-09	1,34E-08	6,13E-09	1,12E-08
Eutrophication, marine	kg N eq	0,00020865	0,00041121	0,00018272	0,00031776	0,00016976	0,00027104
Eutrophication, freshwater	kg P eq	7,10E-05	0,00010112	6,81E-05	8,82E-05	6,67E-05	8,17E-05
Eutrophication, terrestrial	mol N eq	0,00187972	0,00366651	0,00162832	0,00281951	0,00150262	0,00239601
Human toxicity, cancer	CTUh	1,15E-10	2,91E-10	9,71E-11	2,15E-10	8,84E-11	1,77E-10
Human toxicity, non-cancer	CTUh	6,89E-09	9,31E-09	6,61E-09	8,22E-09	6,47E-09	7,68E-09
Ionising radiation	kBq U-235 eq	0,0121531	0,01787694	0,01136897	0,01518487	0,01097691	0,01383883
Land use	Pt	0,80828124	2,2590217	0,65272938	1,6198897	0,57495346	1,3003237
Ozone depletion	kg CFC11 eq	5,54E-09	1,02E-08	4,47E-09	7,59E-09	3,94E-09	6,28E-09
Photochemical ozone formation	kg NMVOC eq	0,00062622	0,00144805	0,00052256	0,00107045	0,00047073	0,00088165
Resource use, fossils	MJ	2,5882208	5,0444364	2,0900204	3,7274975	1,8409203	3,0690281
Resource use, minerals and metals	kg Sb eq	1,15E-05	1,38E-05	1,13E-05	1,28E-05	1,12E-05	1,23E-05
Water use	m3 depriv.	0,07198076	0,11969701	0,06578495	0,09759578	0,06268704	0,08654517

Table S3.8. Sensitivity analyses to test the effect on outcomes of data choices and assumptions

Category	*Explanation*	*Type*	*Source*	*Effect*
**Commute / individual travel**	Dutch average car transportation mix for employees and patients (Ecoinvent) rather than the Netherlands-specific STREAM transportation data	Alternative data choice	main contributing process to Care on Site scenario, largest contributor to RPM scenario after eHealth devices	Alteration influences either nurse commute to patient (main effect) or nurse commute to the office (minor effect, due to allocation). Notable effect on climate change, particulate matter, and fossil resource use (larger for ecoinvent) and water use (larger for STREAM). Increase of e.g. climate change large for CoS (+49%; +0.16kg CO2eq) and minor for RPM (+11%; +0.014kg CO2 eq). Considering the relatively large impact of nurse commute to patient when comparing both scenarios, the choice of Ecoinvent data would favour the choice for videocalling in the reference scenario and would strengthen the effects of the sensitivity analyses (above).
**Energy source**	Dutch average electricity mix (Ecoinvent) rather than the Netherlands-specific energy mix based on CE Delft data (2021): the NL mix contains more fossil fuel based electricity generation	Alternative data choice	minor contributing process in videocalling scenario, known effect of data choice	Alteration only influences videocalling scenario's office energy use (and digital infrastructure for server storage, data transfer, and video calling). Minor effect on climate change, particulate matter, freshwater eutrophication, and fossil resource use. Increase of e.g. climate change for videocalling (+4%; +0.001 kg CO2eq). No substantial effect on comparison of videocalling and CoS scenario.
**Data transfer 'PUE'**	power usage effectiveness (PUE) of data transfer and data storage in kWh/GB is used from Swiss Federal Office for Energy (2020 data adjusted for 2023) or Sillcox et al (2023) rather than the Jackson et al (2023) figures	Alternative data choice	minor contributing proces, verify if impact in videocalling scenario would be substantially larger	Alteration only influences the videocalling scenario. PUEs of respectively 0.0075 kWh/GB (Jackson), 0.113 kWh/GB (SFOE), and 0.64 kWh/GB (Sillcox). Effects on outcomes <1% and therefore no effect on comparison etc.

Note to reader

NB: variance of staff commute and energy use in both Physical Visits and Telemedicine were already included in the uncertainty analysis (Monte Carlo) by predefining possible ranges for the number of patients seen in a day, average surface area of the nursing office rooms, and variations in potential data size (storage) required for usage of videocalling. Therefore no separate sensitivity analysis has been performed.

Figure S3.1. Uncertainty analysis of the Physical Visit (“B”) and Telemedicine (“A”) comparison
Executed in SimaPro v9.5.0.1 using Monte Carlo simulations for 1,000 runs; 95% confidence interval.

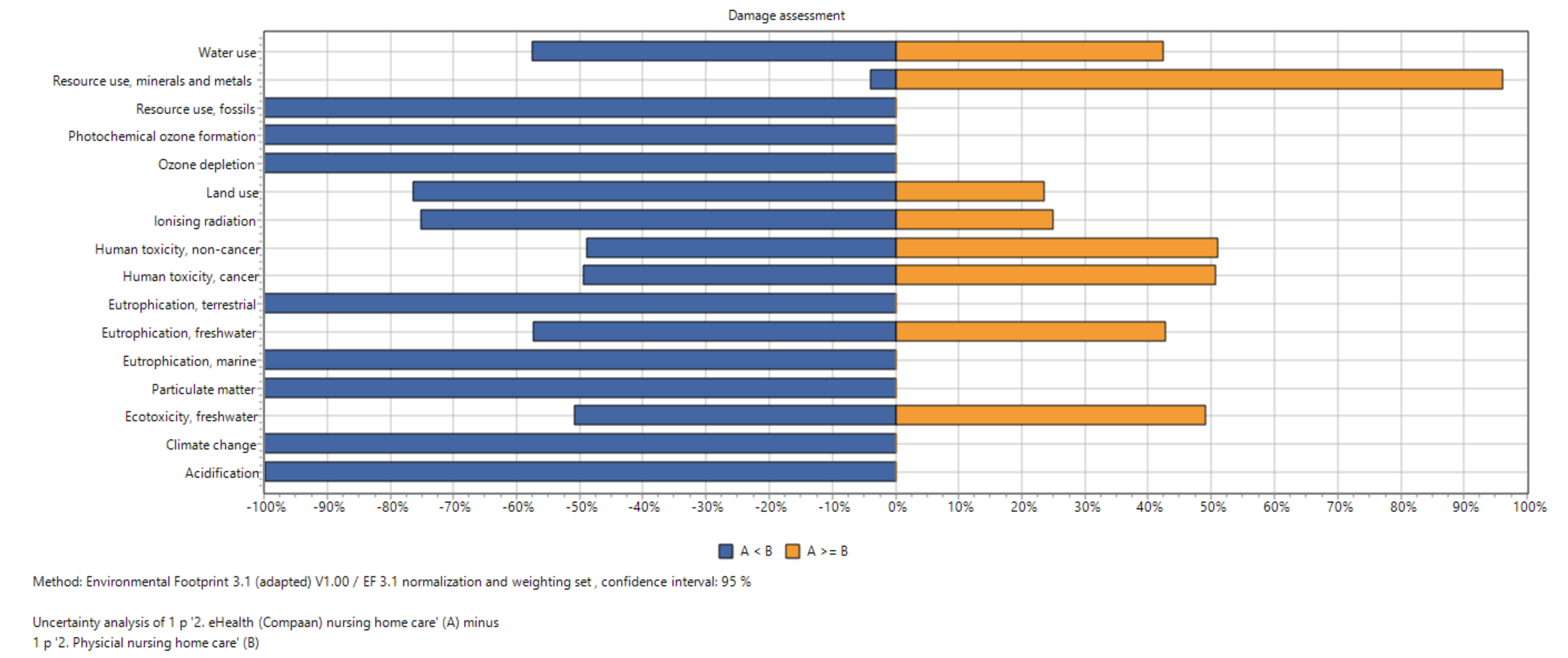

NB: in the comparison above, the staff travel distance between patients for the Physical Visit (“B”) scenario varies between 1 and 3 km (urban setting) and the means of transport is fixed at 80% car travel and 20% bicycle travel.
NB: the Telemedicine (“A”) scenario, the tablet allocation is altered based on the number of calls that is is used (i.e. if it is used for more consecutive days by the same patient, the impact of the tablet is divided over more visits); the redistribution to a second patient is unaltered (since the tablet is always redistributed in the current experience of the telemedicine service company) and the functional allocation is fixed at 100% (methodological choice because patients receive the tablet simply for the telemedicine service – so any personal use will no have environmental impact allocated to it).
NB: for the Telemedicine (“A”) scenario, the staff commute is altered based on the number of patients they see per day (20-40; since the impact of their commute is divided over the different patients) and the travel distance (4-20km); the percentage working from home remains fixed (50%).
NB: for the Telemedicine (“A”) scenario, the office energy use is altered based on the number of patients and office surface area.

Note: no uncertainty analysis for the comparison has been made for the ‘rural’ scenario wherein the distance between patients is substantially longer (5-15km), considering that the sensitivity analysis already indicates that longer staff travel distances favor the use of Telemedicine.

References

1. Arduin RH, Charbuillet C, Berthoud F, Perry N. Life cycle assessment of end-of-life scenarios tablet case study. Conference: Sardinia 2017 - 16th Waste Management and Landfill Symposium.
2. Güvendik M. From smartphone to futurephone: assessing the environmental impacts of different circular economy scenarios of a smartphone using LCA. (2014) Available via: https://repository.tudelft.nl/record/uuid:13c85c95-cf75-43d2-bb61-ee8cf0acf4ff
3. Berg, van den R, Seters, van D. STREAM personenvervoer 2023. Delft: *CE Delft*; 2024.
4. Kleijn A, Hilster D, Otten M, Scholten P. STREAM Freight Transport 2020. Delft: *CE Delft*; 2021.
5. Milieubarometer. Kantoor – branchegemiddelde. Rotterdam: *Stichting Stimular*; 2022.
6. Bruinsma M, Nauta M. Ketenemissies elektriciteit: Acutalisatie elektriciteitsmix 2021. Delft: *CE Delft*; 2023.
7. Thiel CL, Mehta N, Sejo CS, et al. Telemedicine and the environment: life cycle environmental emissions from in-person and virtual clinic visits. *NPJ Digit Med.* 2023;**6**(1):87.
8. Jamal H, Lyne A, Ashley P, Duane B. Non-sterile examination gloves and sterile surgical gloves: which are more sustainable? *J Hosp Infect.* 2021;**118**:87-95.
9. Rizan C, Reed M, Bhutta MF. Environmental impact of personal protective equipment distributed for use by health and social care services in England in the first six months of the COVID-19 pandemic. *J R Soc Med.* 2021;**114**(5):250-63
10. Jackson T, Hodgkinson IR. Is there a role for knowledge management in saving the planet from too much data? *Knowl Manag Res Pract.* 2023;**21**(3):427–35
11. Coroama V. Investigating the Inconsistencies among Energy and Energy Intensity Estimates of the Internet. Bern: *Swiss Federal Office of Energy SFOE*; 2021.
12. Koho Enterprise Solid-State Drive Product Life Cycle Assessment Summary. USA: *Seagate Technology LLC*; 2016.
13. Tannu S, Nair PJ. The Dirty Secret of SSDs: Embodied Carbon. ACM SIGENERGY Energy Inf Rev. 2023;**3**(3):4–9
